# Kinematic analyses including finite helical axes of drop jump landings demonstrate decreased knee control long after anterior cruciate ligament injury

**DOI:** 10.1371/journal.pone.0224261

**Published:** 2019-10-31

**Authors:** Helena Grip, Eva Tengman, Dario G. Liebermann, Charlotte K. Häger

**Affiliations:** 1 Department of Radiation Sciences, Biomedical Engineering, Umeå University, Umeå, Sweden; 2 Department of Community Medicine and Rehabilitation, Physiotherapy, Umeå University, Umeå, Sweden; 3 Department of Physiotherapy, Stanley Steyer School of Health Professions, Sackler Faculty of Medicine, Tel Aviv University, Tel Aviv, Israel; University of Memphis, UNITED STATES

## Abstract

The purpose was to evaluate the dynamic knee control during a drop jump test following injury of the anterior cruciate ligament injury (ACL) using finite helical axes. Persons injured 17–28 years ago, treated with either physiotherapy (ACLPT, n = 23) or reconstruction and physiotherapy (ACLR, n = 28) and asymptomatic controls (CTRL, n = 22) performed a drop jump test, while kinematics were registered by motion capture. We analysed the *Preparation* phase (from maximal knee extension during flight until 50 ms post-touchdown) followed by an *Action* phase (until maximal knee flexion post-touchdown). Range of knee motion (*RoM*), and the length of each phase (*Duration)* were computed. The finite knee helical axis was analysed for momentary intervals of ~15° of knee motion by its intersection (*ΔAP position*) and inclination (*ΔAP Inclination*) with the knee’s Anterior-Posterior (AP) axis. Static knee laxity (KT100) and self-reported knee function (Lysholm score) were also assessed. The results showed that both phases were shorter for the ACL groups compared to controls (CTRL-ACLR: *Duration* 35±8 ms, p = 0.000, CTRL-ACLPT: 33±9 ms, p = 0.000) and involved less knee flexion (CTRL-ACLR: *RoM* 6.6±1.9°, p = 0.002, CTRL-ACLR: 7.5 ±2.0°, p = 0.001). Low *RoM* and *Duration* correlated significantly with worse knee function according to Lysholm and higher knee laxity according to KT-1000. Three finite helical axes were analysed. The *ΔAP position* for the first axis was most anterior in ACLPT compared to ACLR (*ΔAP position* -1, ACLPT-ACLR: 13±3 mm, p = 0.004), with correlations to KT-1000 (rho 0.316, p = 0.008), while the *ΔAP inclination* for the third axis was smaller in the ACLPT group compared to controls (*ΔAP inclination* -3 ACLPT-CTRL: -13±5°, p = 0.004) and showed a significant side difference in ACL injured groups during *Action* (Injured-Non-injured: 8±2.7°, p = 0.006). Small *ΔAP inclination* -3 correlated with low Lysholm (rho 0.391, p = 0.002) and high KT-1000 (rho -0.450, p = 0.001).

*Conclusions* Compensatory movement strategies seem to be used to protect the injured knee during landing. A decreased *ΔAP inclination* in injured knees during *Action* suggests that the dynamic knee control may remain compromised even long after injury.

## Introduction

Rupture of the anterior cruciate ligament (ACL) is a common sports injury that most often occurs due to high strain on the ACL when load forces are multi-directional, such as when the knee is abducted and internally rotated [1]. Since the ACL plays a major role in stabilising the knee [1], its rupture results in loss of knee joint control in weight-bearing conditions and leads to muscle weakness and sensorimotor impairment [2].

Intervention strategies include physiotherapy (PT) alone or in combination with reconstructive surgery. It is debated how the different interventions influence knee stability and the risk of knee osteoarthritis (OA) development in the long term. An 11-year follow-up on 109 persons treated with reconstruction versus PT showed higher stability and higher degree of OA after ACL reconstruction, whereby the result is not necessarily perceived as better subjectively [3]. A retrospective study on 54 patients treated with reconstruction or PT on the other hand showed that 94% of operated patients had stable knees after 15–20 years with a lower percentage of OA in comparison to conservatively treated patients with 84% having abnormal or severe laxity [4]. Regardless of treatment strategy, effective rehabilitation and training is needed to improve neuromuscular control of the knee after injury [5].

To specifically address dynamic valgus and sagittal plane movements, a helical (screw) axis parameterisation may be used. With this method dynamic changes in knee motion direction and joint configuration can be analysed by the momentary changes in inclination and position of the knee helical axis, HA [6]. We recently used such a parameterisation to show that the knee helical axis is positioned differently during a relatively controlled task (squat) compared to a more knee-challenging task (side hop) and particularly in 70 persons who had suffered an ACL injury 17–28 years previously (treated with surgery, n = 33, or PT, n = 37) compared to 33 age- and sex-matched asymptomatic controls [7]. In another recent study of ours on partly the same study population, curve analyses of trunk, hip and knee joints during a drop jump test indicated compensatory movement patterns 17–28 years after an ACL injury [8]. We now want to specifically address dynamic knee valgus and sagittal plane movements in this study group by analysing the knee HA during the landing phase of a drop jump test. The test involves a vertical drop jump from a platform, landing on both legs simultaneously, immediately followed by a vertical jump, and is frequently used to evaluate neuromuscular control of the knee in injury prevention and rehabilitation. Real-time video-based observational screening has been used to identify athletes with high knee valgus angles in the vertical drop jump landing [9], even though it has been questioned that this approach can be used to predict ACL injuries [10]. Meyer and colleagues used the drop jump test to show that following surgery of an ACL injured knee, patients employ a knee unloading strategy of their injured leg giving less sagittal knee power/energy absorption during the landing, something that is important to correct to avoid second ACL injuries or early-onset OA development [11]. A recent Delphi panel study with 20 invited clinicians and researchers identified three important outcomes of the vertical drop jump test during rehabilitation after ACL injury; knee valgus collapse (no collapse to extreme collapse), the presence of other undesirable movements (lateral trunk lean, insufficient knee flexion) and limb-to-limb asymmetry [12].

The aim of this study was to observe dynamic knee control during the drop jump test in people long term after ACL injury compared to asymptomatic controls. For this purpose, we used a finite helical axis analysis in the preparation phase prior to the landing and the landing phase following a vertical drop jump. We also computed the range of knee motion and phase duration to analyse landing strategy. We hypothesised that ACL-injured persons would unload and protect their injured knee during preparation [11] and that excessive abduction-adduction movements and tibial anterior-posterior (AP) translations would occur in the ACL-injured knee during landing [12]. We also expected persons treated with reconstruction combined with PT to have a greater dynamic knee stability more resembling those observed in asymptomatic controls, in comparison to persons treated only with PT [3, 4].

## Materials and methods

### Participants

**Fig 1:**
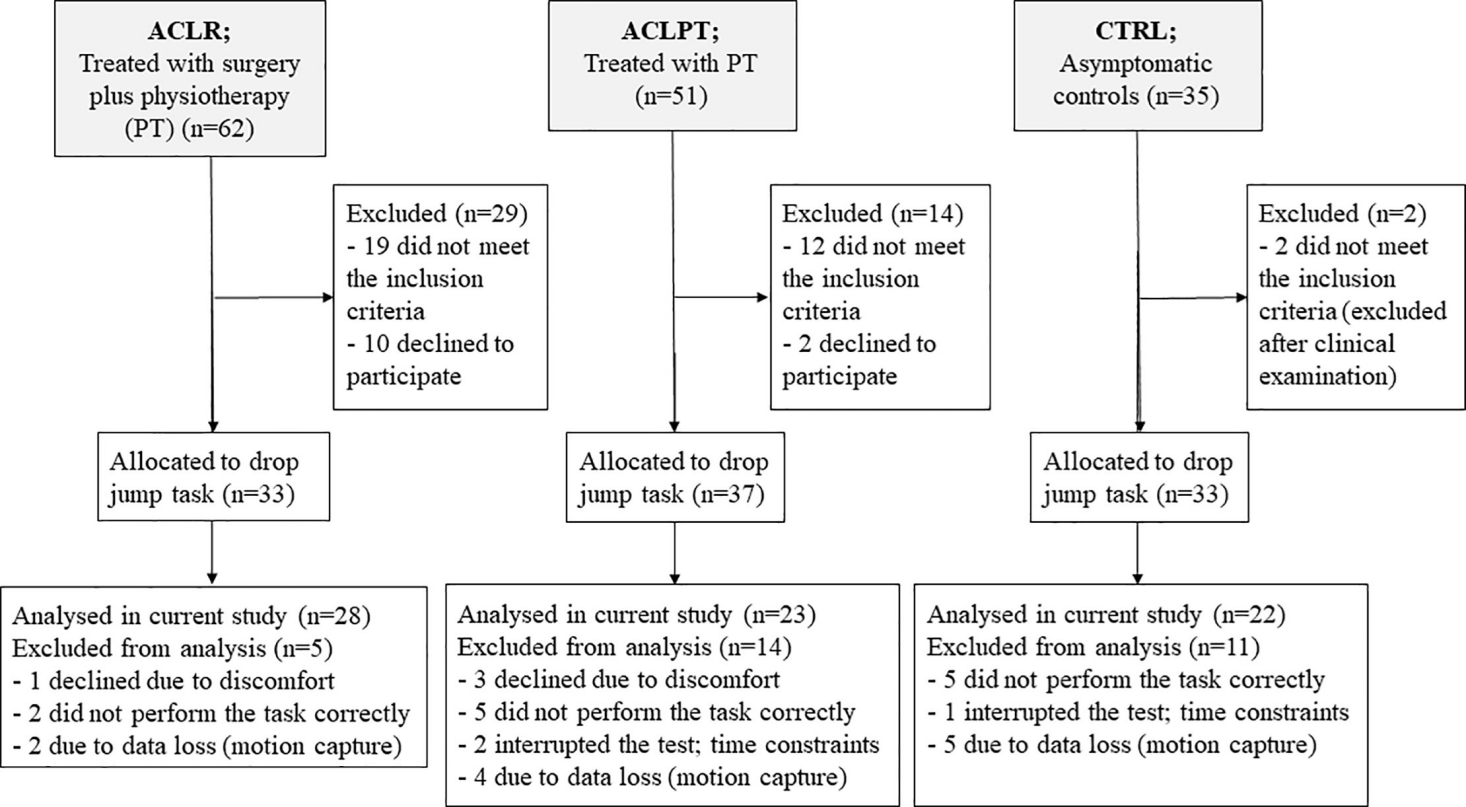
Participant recruitment flow diagram. The flow diagram describes how the participants were recruited and selected to the drop jump test.

This retrospective cohort study is part of a larger cross-sectional research program involving two cohorts from two different hospitals in two county councils in northern Sweden that in total consisted of 148 individuals: 113 individuals who sustained an ACL injury which occurred 17–28 years prior to this follow-up and 35 asymptomatic controls (KACL20-study). The KACL20-study included studies on knee function during different functional tasks such as stair gait, one-leg hop tests and strength tests as reported previously, e.g. [7, 8, 13]. Seventy-three participants (51 individuals with ACL injury and 22 controls) were analysed in the current study as described in the flow chart (Fig 1).

The ACL reconstructed group, ACLR, consisted of 33 participants that were injured between 1981 and 1993 and then treated with reconstructive surgery followed by post-operative PT. Nineteen participants received a patellar tendon autograft augmented with a synthetic polypropylene braid placed over the top, nine participants had a Kennedy Ligament Augmentation Device (LAD) graft placed through a femoral tunnel and five participants received a bone-patellar tendon-bone graft. The post-operative PT aimed to regain full knee range of motion and improve leg strength, coordination, and balance. The conservatively treated group, ACLPT, consisted of 37 persons injured between 1993 and 1998. This cohort was treated with PT alone, consisting of a tailored goal-oriented rehabilitation programme designed by the physiotherapist and the orthopaedic surgeon in charge. Exclusion criteria for ACLPT and ACLR were knee prosthesis surgery, bilateral injuries, multiple joint structural damage in addition to the ACL injury, or other musculoskeletal, rheumatologic, and/or neurological pathologies that might influence the participant’s movement ability. The control group, CTRL, consisted of 33 age- and sex-matched controls with asymptomatic knees, hips and feet, recruited via advertisements and convenience sampling via word of mouth. Controls reported no previous knee injuries, as verified by clinical examination, questionnaires and interviews.

Thirty participants were excluded from the analyses for the following reasons: three participants interrupted the test due to time constraints (the drop jump task was last in a test protocol that took ~two hours to perform), four participants did not perform the task as they were apprehensive about the landing, 11 were excluded due to data loss and 12 participants were not able to perform a correct drop jump (see testing procedure). Hence, 73 participants (28 ACLR, 23 ACLPT and 22 CTRL) were included for kinematic analyses. The demographic data of these participants is presented in Table 1. All participants were given written and oral information about the study and gave their written informed consent according to the declaration of Helsinki. The study was approved by the Regional Ethical Review Board in Umeå, Sweden.

**Table 1:** Demographic data. Demographic data of the participants; ACL-injured participant, treated with surgery and physical therapy (ACLR), ACL-injured participant, treated with physical therapy (ACLPT) and healthy-knee controls (CTRL). Group mean values are given as Mean (SD).

	ACLR	ACLPT	CTRL
Number of participants	28	23	22
Women/Men	8/20	9/14	9/13
Body Mass Index (kg/m^2^)	27.4 (3.5)	27.2 (3.7)	24.7 (2.6)
Years since injury	23.1 (2.9)	22.1 (1.1)	-
Lysholm score	81.1 (17.2)	71.5 (12.8)	-
Tegner activity scale	4.5 (1.3)	4.4 (1.4)	6.2 (1.3)
KT-1000 (mm); side difference	^§^1.9 (2.8)	^§^5.0 (3.1)	^#^ 0.2 (1.1)
Case of injury			
Soccer Other sports	21 7	17 4	
Non-sporting	None	2	

^§^ Non-injured–Injured

^#^ Dominant–Non-dominant

### Test protocol

**Fig 2:**
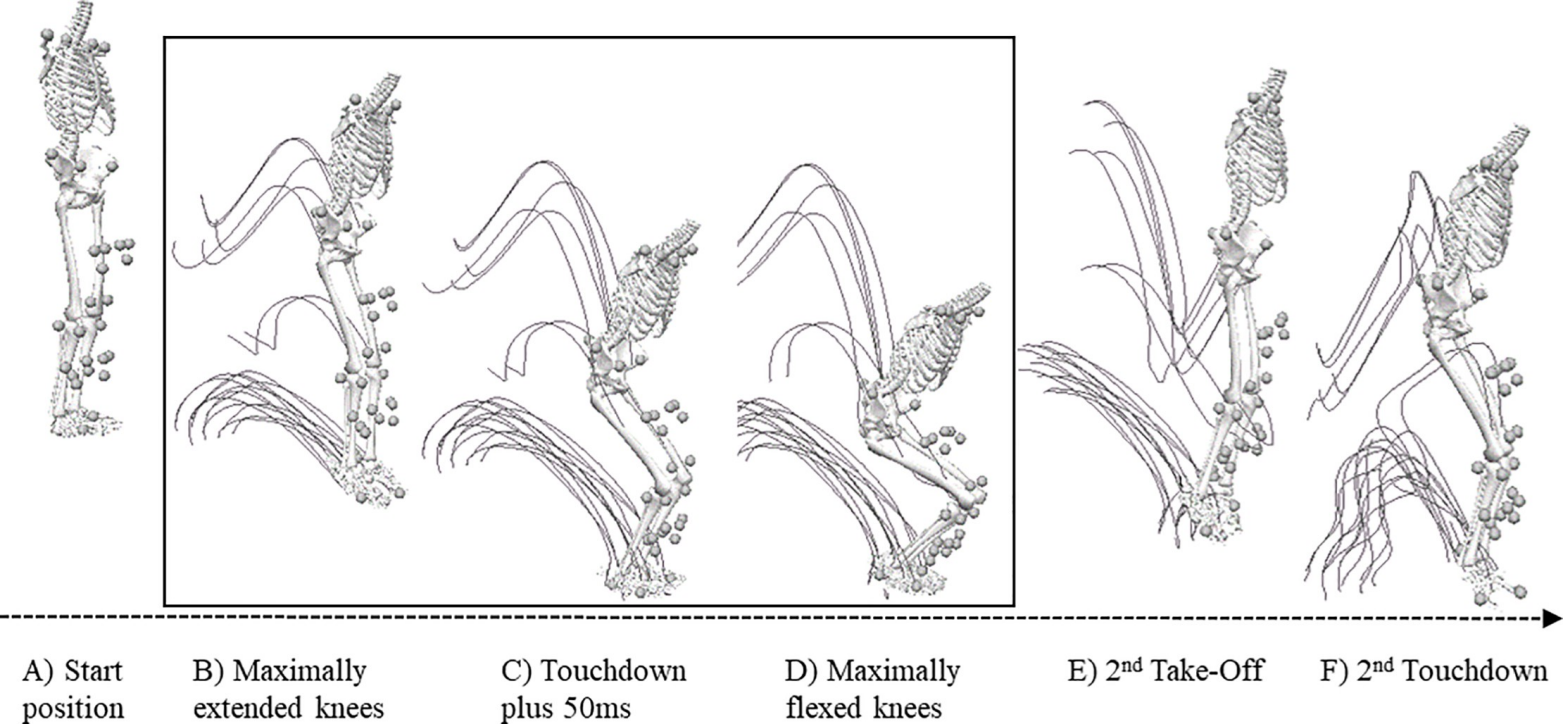
The vertical drop jump test. Illustration of one asymptomatic control performing the vertical drop jump test. Each participant was instructed to jump from a 0.4 m high platform and was instructed to land on both legs simultaneously. A vertical jump followed immediately after the first landing. The first landing sequence (marked by a square) was analysed in this study, and was divided into a *Preparation* phase, which started after the take-off when the person had maximally extended knees, followed immediately by an *Action* phase, which ended when the person reached their maximum knee flexion angle.

Each participant completed a self-rated knee function questionnaire, the Lysholm score (0 to 100, where “100” represents excellent knee function) [14], and the Tegner activity scale that grades physical activity level (0 to 10, where “10” represents participation in national and international elite competitive sports). A physiotherapist performed a static knee laxity test using a KT-1000 arthrometer that measured anterior tibial translation while applying a pulling force of 30 lb [15]. The drop jump test was then performed in a movement laboratory to investigate dynamic knee stability. Since this test was part of a larger test battery, each participant started with a 6-minute warm-up on a bicycle ergometer at a moderate intensity before performing gait, balance, strength and hop tests. These other tests were analysed and reported previously e.g. (7, 8, 13). The drop jump took place last in the test battery and started with the participant standing on a 0.4 m high platform with the arms relaxed at either side of the body. Arm motion was unconstrained during the rest of the test, which consisted of a vertical drop jump, landing on both legs, followed by an immediate upward jump that ended with a second landing. The first drop landing was analysed in the current study (Fig 2). The drop jump test was defined as successful if the person was able to perform the second jump without jumping excessively forwards and without losing balance (for example needed to take an extra step to restore balance prior to the second jump). Persons that failed to perform a successful test were excluded from further analyses.

### Data collection

3D movement data were collected during the drop jump test using an electro-optical motion capture system (Oqus, Qualisys Medical AB®, Gothenburg, Sweden; 240 Hz, 8 cameras) that collected coordinate data from spherical passive reflective markers placed on the body. The markers (12 mm and 19 mm in diameter) were affixed with double-sided adhesive tape to the skin on pre-defined anatomical landmarks. The anatomical positions used for the kinematic analyses in this study were: two on the shoulders (one each acromion), five on the pelvis (one placed on sacrum, one on each ilac crest and each anterior superior iliac spine), one on each greater trochanter, two on each knee joint (lateral and medial epicondyles), three on each shank (one on the tuberositas tibiae and one on the lateral and medial malleoli), and 2 on each foot (at the distal metatarsals on the lateral and medial side of the foot). In addition, rigid plates with cluster of three markers were attached with elastic straps and adhesive tape to each shank and thigh to reduce measurement errors caused by soft tissue artefacts. This resulted in a total of 35 markers used for model construction and joint angular calculations. Marker trajectories were identified using the QTM software 2.11 (Qualisys Medical AB®, Gothenburg, Sweden) and scrutinized by visual inspection to decide whether interpolation of momentarily hidden marker trajectories was required, using a maximum number of 10 frames (40 ms) for gap-filling.

### Definition of the kinematic model and outcome measures

Raw marker data were pre-processed using a second-order low-pass Butterworth 15 Hz filter with zero-lag (i.e., ran twice in reversed order) and a 6-degrees-of-freedom segment model was computed using the Visual3D package (v.5.02.30, C-Motion Inc. Germantown, MD, USA). A 6-degree-of-freedom kinematic segment model including the upper body, pelvis, legs (thigh and shank) and feet were constructed (Fig 2). For all segments, +X represented the mediolateral (ML) axis, +Y represented the anteroposterior (AP) axis and +Z the vertical axis [16]. The shank coordinate system was defined from the markers on the medial/lateral knee condyles and medial/lateral malleoli, while the thigh coordinate system was defined from markers on the sacrum, trochanters and medial/lateral knee joints [17]. The knee joint angle was defined from the rotation of the shank relative to the thigh using the Cardan XYZ sequence.

Three events were used to define the phases: *Maximal knee extension*; the point in time when knees were maximally extended during the flight after the vertical drop jump, *Touchdown*; taken as the instance when the vertical velocity of the foot marker was less than 0.2 m/s and *Maximal knee flexion*; the point in time when knees were maximally flexed after *Touchdown* (Fig 2). The events were detected automatically by an algorithm according to these definitions and were then scrutinized and verified by inspection using segmental models and motion curves in Visual3D.

**Fig 3:**
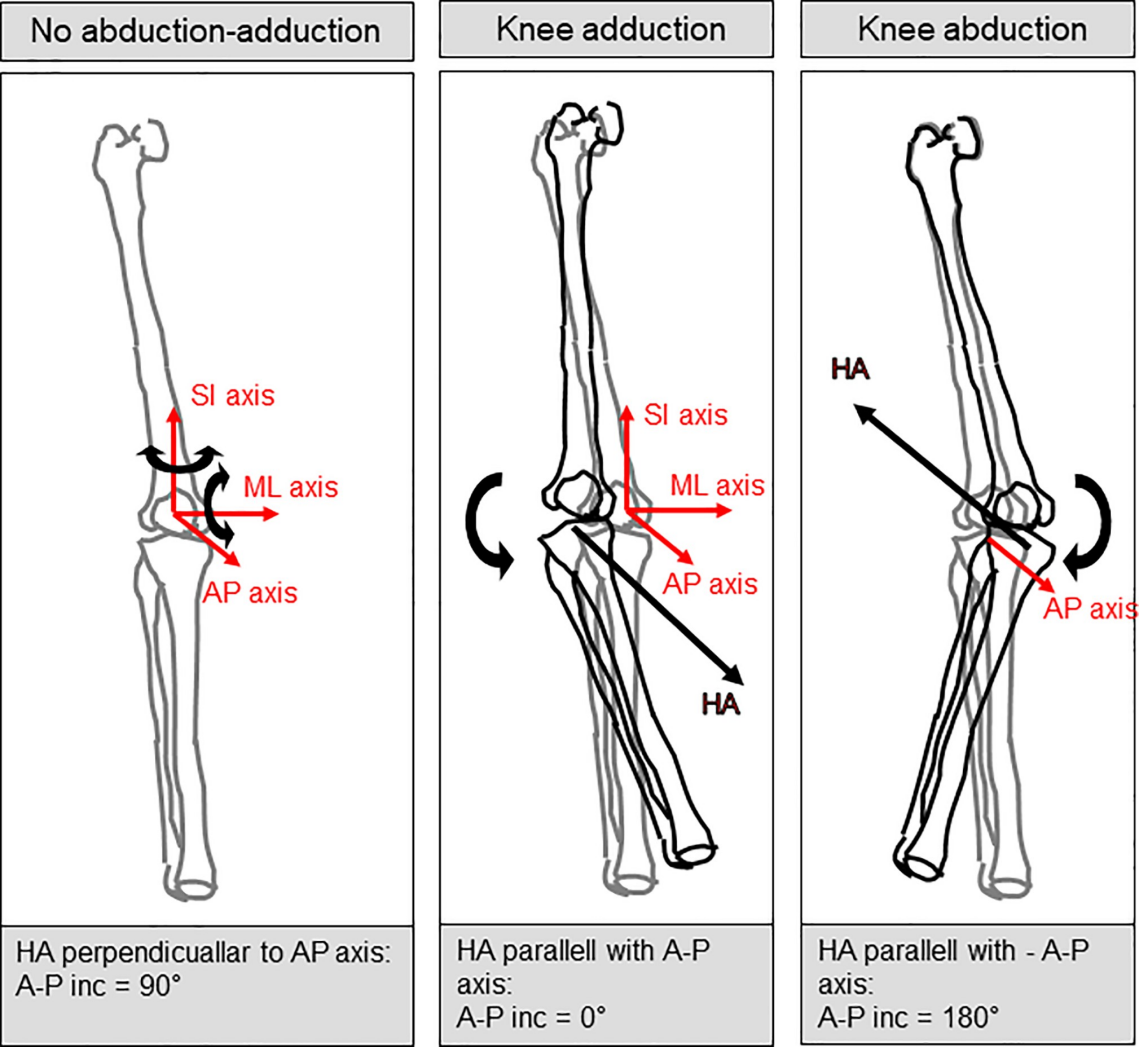
Description of helical axis variables. The 3D angle between the finite helical axis (FHA) of the knee during a movement interval of ~15° and the AP axis of the knee illustrate the relationship between the magnitude of abduction-adduction relative to magnitude of movement in other planes (flexion-extension and inward-outward rotation). Hence if the ΔAP inclination is 90°, the FHA is normal to the AP axis and the knee motion occurs in the sagittal and/or transverse planes. An inclination of 0–90° means adduction occurs (the smaller number the more adduction in relation to other knee movement), while an inclination of 90–180° indicates that abduction occurs (the larger number the more abduction in relation to other knee movements).

Two phases following the vertical drop jump were analysed. During the flight after the vertical drop jump, leg extensors and flexors were expected to activate in order to stiffen the knee joint for up to 50 ms after touchdown [18]. This phase was in our study referred to as *Preparation* and was defined to start at *Maximal knee extension* and stop 50 ms after *Touchdown*. After *Preparation*, a phase of active knee joint control follows, when eccentric and concentric muscular activity is expected to reduce the impact on the knee joint [18]. This phase was referred to as *Action* in this study and was defined to start 50 ms after *Touchdown* and ending at *Maximum knee flexion* (Fig 2). Kinematic measures were computed for each landing phase using MatLab (ver. 8.0.0.783, R2012b, MathWorks Inc., Natick, MA, USA). The range of motion (*RoM*) in knee flexion-extension (FE) and abduction-adduction (AbAdd) and the duration of each phase (*Duration*) were computed to analyse landing strategy. The momentary (finite) helical axis of the knee (FHA) was computed for discrete intervals of ~15° knee angular motion (whether flexion-extension, abduction-adduction and/or inward/outward rotation), a limit which was based on error simulations in a previous study [17]. The exact interval length (Δ) was adjusted with a tolerance level of 70% so that a maximal number of intervals could be achieved for each person and each phase. This means that each interval could be between 10.5–15°. To assess movement direction; i.e. the amount of angular motion around the AP axis (abduction—adduction) in relation to angular knee motion in the two other planes (flexion-extension and inward-outward rotation), we calculated the 3D angle between the FHA and the knee’s AP axis for each interval. As illustrated in Fig 3, *ΔAP inclination* < 90 means that knee adduction occurs, while *ΔAP inclination* > 90 means that knee abduction. An *ΔAP inclination* of exactly 90° occurs when the helical axis is normal to the knee’s AP axis and represents knee movement solely in the sagittal and/or transverse planes. To assess translations in the tibial AP direction, the position of the FHA relative to the distal end of the thigh in the AP direction was computed. This variable was named Δ*AP position* and was determined by first calculating the point of intersection of the FHA vector with the knee’s mid-sagittal plane. The midline of the knee was defined in the segment model as the line between the markers on the medial and lateral epicondyles. The projection of this position on the shank’s AP axis defined the Δ*AP position*.

### Statistical analyses

The software package SPSS (ver. 22.0.0, IBM; Chicago, IL, USA) was used for all statistical analyses. Injured knees were compared to asymptomatic controls (group comparisons) and to contralateral knees (side comparisons). Injured knees were compared to non-dominant control knees (this choice is considered more stringent since the non-injured leg often becomes the preferred leg after an injury). The dominant leg was defined as the leg self-preferred to kick a ball.

In this study, dynamic knee control was analysed in people a long term after an ACL injury compared to asymptomatic controls. Side differences within each ACL group were also addressed. The statistical models comprise analyses of two types of variables: (1) *RoM* and *Duration* which are single parameters and (2) a series of FHA inclination angle and position values (*ΔAP inclination* and *ΔAP position*), which varied in number across individuals and phases. This resulted in four different statistical models as described below.

### Group comparisons of healthy controls and ACL-injured legs

Group comparisons of kinematic and temporal parameters (*RoM-FE*, *RoM-AbAdd*, *Duration*, *ΔAP inclination* and *ΔAP position)* between injured knees and control knees were performed by linear mixed model designs. The number of FHA’s depends on the individual’s knee *RoM*. Only FHA variables that existed for >75% of participants in each group were analysed. In each model, “Group” (ACLR, ACLPT and CTRL), “Phase” (*Preparation* and *Action*) and the interaction Group × Phase was included as fixed effects. “Participant” was included as a random effect. This effect was significant in all models. Adjusted Bonferroni post-hoc analyses were used to assess pair-wise effects for significant factors and interactions. *F* statistics, exact *p*-values and 95% confidence intervals (CI) are reported.

To explore the relationships between the kinematic variables during each phase in more detail, a Principal Component Analysis (PCA) was performed for each phase. Variables of significant importance (i.e., variables that differed significantly between injured and healthy knees) were included and Principal components (PC’s) with eigenvalues > 1 were extracted to assess significant variable’s contributions to the total data covariance matrix.

### Side comparisons within ACL groups

Side comparisons of kinematic and temporal parameters (*RoM-FE*, *RoM-AbAdd*, *Duration*, *ΔAP inclination* and *ΔAP position)* between the injured and non-injured leg were analysed with linear mixed models for each outcome variable and phase independently. The model was set up as the previously described for group comparisons, except that “Side” (injured vs. non-injured) and “Group” (ACLR vs. ACLPT) and the interaction “Group×Side were included as fixed effects. *F* statistics, exact *p*-values and 95% confidence intervals (CI) for side comparisons are reported if significant.

The kinematic measures that differed significantly between the ACL groups and/or sides were further explored by correlation analyses. The Spearman’s rank correlation coefficient was used to test the relationship between kinematic measures and clinical measures (static knee laxity graded by KT-1000 arthrometer and knee function graded by Lysholm score) for the injured leg.

## Results

**Fig 4:**
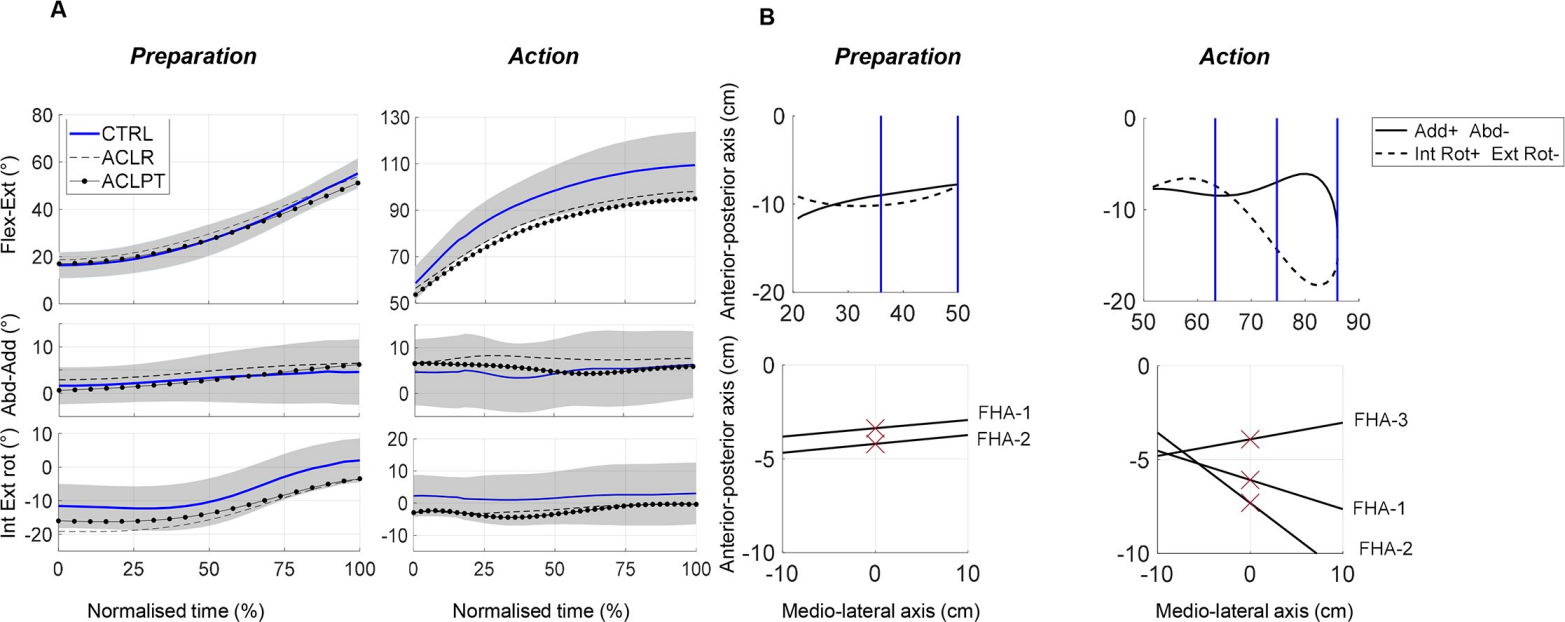
**A-B. Knee joint motion during the vertical drop jump test.** Fig 4A illustrates the knee joint motion for the *Preparation* and *Action* phases after a vertical drop jump, averaged for each group. Group mean curves given for non-dominant knees of CTRL (thick black line, plus standard deviation shaded in grey), ACLR injured knees (dashed line) and ACLPT injured knees (dotted line). In Fig 4B, the knee joint movement of an ACLPT participant’s injured knee during *Preparation* and *Action* is illustrated by angle-angle diagrams (upper row) and knee helical axes (bottom row). The vertical lines in the angle-angle diagrams mark the flexion intervals that were used when computing the FHA’s. The vertical line in the helical axis plots marks the thigh’s mid-sagittal plane. This participant had an almost constant Δ*AP inclination* during the *Preparation* phase (Δ*AP inclination* about 84°). During the *Action* phase however, the Δ*AP inclination* ranged from 80 to 124°.

Fig 4A illustrates group mean angle-time curves during *Preparation* and *Action*, highlighting that on average the knees flexed, rotated internally and adducted during *Preparation* in all groups and continued to flex during *Action*, while remaining in adduction, which indicates that no extreme knee valgus collapse occurred in any of the groups. An example of joint angles and resulting ΔHA’s for one of the ACL-injured participants is displayed in Fig 4B.

### Knee kinematics during preparation

In comparison to healthy knees, the injured knees of ACLR and ACLPT persons had smaller *RoM-FE* and shorter *Duration*, while no differences were found in *RoM-AbAdd (*Table 2). Correlation analyses showed that shorter *Duration* correlated with larger knee laxity according to KT-1000 (rho -0.307, p = 0.034) and lower self-reported knee function according to the Lysholm score (rho 0.222, p = 0.025), while smaller *RoM-FE* correlated to a lower Lysholm score (rho 0.452, p = 0.001).

**Table 2:** Knee range of motion and duration when landing after a vertical drop jump.

Variable	CTRL	ACLR	ACLPT	F statistic (*p* value)	Post hoc (*p* value)
*ROM-FE (°)*				*Grp*: *F*(2, 70) = 8.7 (.000)	CTRL > ACLR (.002)
*Preparation*	36.5–43.6	32.6–38.9	31.3–38.2	*Phase*: *F*(1, 70) = 38.7 (.000)	CTRL > ACLPT (.001)
*Action*	47.0–54.1	38.5–44.7	37.5–44.4	*Grp×Phase*: *F*(2, 70) = 1.5 (.231)	ACLR vs. ACLPT (1.00)
*ROM-AbAdd (°)*				*Grp*: *F*(2, 70) = 0.5 (.585)	
*Preparation*	4.0–7.3	4.0–6.8	4.4–7.6	*Phase*: *F*(1, 70) = 23.6 (.000)	
*Action*	6.4–9.7	6.6–9.5	7.5–10.7	*Grp×Phase*: *F*(2, 70) = 0.1 (.887)	
*Duration (ms)*				*Grp*: *F*(2, 140) = 10.9 (.000)	CTRL > ACLR (*Prep*: .029, *Act*: .000)
*Preparation*	103–137	90–121	86–119	*Phase*: *F*(1, 140) = 156.6 (.000)	CTRL > ACLPT (*Prep*: .012, *Act*: .003)
*Action*	211–245	158–188	163–197	*Grp×Phase*: *F*(2, 140) = 3.3 (.040)	ACLR vs. ACLPT (*Prep*: .646, *Act*: .623)

* The range of motion in flexion-extension (*ROM-FE*), the range of motion in abduction-adduction (*ROM-AbAdd*) and *Duration* were analysed during the *Preparation* and *Action* phases following a vertical drop jump.

^¤^Confidence intervals of 95% of adjusted marginal means are given for each group.

^#^ The *F* statistics (between-groups and within-groups degrees of freedom within parentheses) and *p* value are reported for fixed effects and interactions.

^$^ Post hoc pairwise comparisons for significant group effects and interactions are given.

Three *FHAs* were analysed. During *Preparation*, the number of *FHA*s were CTRL = 2.7 ± 0.5; ACLR = 2.4 ± 0.6; ACLPT; 2.2 ± 0.4. The *ΔAP inclination* was < 90°, which means that adduction occurred while the knee flexed/rotated. It was smaller in ACLPT compared to controls toward the end of the phase (Table 3). The *ΔAP position* was mainly posterior relative to the knee joint centre in all groups. It was significantly greater (more anterior) in ACLPT compared to the ACLR in the beginning of the phase (Table 3). A greater *ΔAP position-1* also correlated to greater static knee laxity according to KT-1000 (0.316, p = 0.008).

**Table 3:** Knee helical axis variables when landing after a vertical drop jump.

Variable*	CTRL^¤^	ACLR^¤^	ACLPT^¤^	F statistic (*p* value) ^#^	Post hoc (*p* value)^$^
*ΔAP Inc -1 (°)*				*Group*: *F*(2, 69) = 1.3 (.270)	
*Preparation*	75.0–85.0	69.4–78.2	71.5–81.3	*Phase*: *F*(1, 69) = 87.1 (.000)	
*Action*	86.1–96.3	84.7–93.5	88.6–98.4	*Group×Phase*: *F*(2, 69) = 1.1 (.327)	
*ΔAP Inc -2 (°)*				*Grp*: *F*(2, 70) = 0.5 (.615)	
*Preparation*	65.1–74.8	64.8–73.4	63.1–72.5	*Phase*: *F*(1, 70) = 171.7 (.000)	
*Action*	86.6–96.6	83.5–92.4	88.6–98.1	*Grp×Phase*: *F*(2, 70) = 1.4 (.244)	
*ΔAP Inc -3 (°)*				*Grp*: *F*(2, 68) = 4.5 (.015)	CTRL vs. ACLR (.054)
*Preparation*	71.8–85.2	63.8–78.4	55.5–81.3	*Phase*: *F*(1, 31) = 13.7 (.000)	CTRL > ACLPT (.036)
*Action*	85.0–97.3	73.8–85.3	70.4–82.7	*Grp×Phase*: *F*(2, 30) = 0.4 (.657)	ACLR vs. ACLPT (1.00)
*ΔAP Pos -1 (°)*				*Grp*: *F*(2, 70) = 6.4 (.003)	CTRL vs. ACLR (.957)
*Preparation*	-9.1–4.0	-17.0 - -5.3	-3.9–9.1	*Phase*: *F*(1, 70) = 5.6 (.021)	CTRL vs. ACLPT (.064)
*Action*	-20.3–6.8	-18.1 –-6.4	-7.5–5.4	*Grp×Phase*: *F*(2, 70) = 1.8 (.181)	ACLR < ACLPT (.002)
*ΔAP Pos -2 (°)*				*Grp*: *F*(2, 69) = 4.8 (.011)	CTRL vs. ACLR (.426)
*Preparation*	-2.9–12.0	-10.1–3.2	0.1–14.7	*Phase*: *F*(1, 68) = 59.4 (.000)	CTRL vs. ACLPT (.414)
*Action*	-24.2 - -9.0	-28.2 - -14.5	-13.5–1.1	*Grp×Phase*: *F*(2, 68) = 0.9 (.428)	ACLR < ACLPT (.008)
*ΔAP Pos -3 (°)*				*Grp*: *F*(2, 70) = 1.4 (.262)	
*Preparation*	-6.7–15.7	1.4–26.2	-2.0–43	*Phase*: *F*(1, 42) = 10.2 (.003)	
*Action*	-17.0–3.3	-12.0–6.8	-8.0–12.0	*Grp×Phase*: *F*(2, 41) = 0.2 (.782)	

* The variables *ΔAP inclination* (mm) and *ΔAP position* (°) were analysed for three consecutive intervals during *Preparation* and *Action*.

^¤^Confidence intervals of 95% of adjusted marginal means are given for each group.

^#^ The *F* statistics (between-groups and within-groups degrees of freedom within parentheses) and *p* value are reported for fixed effects and interactions.

^$^ Post hoc pairwise comparisons for significant group effects and interactions are given.

Side comparisons of the injured and non-injured knee within the ACL groups showed a significant interaction Grp×Side for *Duration* (F (1, 49) = 7.8, p = 0.007), revealing that this phase lasted longer in the injured knee of the ACLPT group (95% CI 94–111 ms in the injured side compared to 88–105 ms in the non-injured).

### Knee kinematics during action

The injured knees of ACLR and ACLPT had a shorter *Duration* and smaller *RoM-FE* compared to controls during the *Action* phase (Table 2), and smaller *RoM-FE* correlated with lower self-reported knee function according to the Lysholm score (rho 0.222, p = 0.012). Three Δ*HAs* existed for > 75% of participants in each group (number of HAs: CTRL = 3.3 ± 1.1; ACLR = 3.0 ± 1.0; ACLPT = 3.0 ± 0.6). The Δ*AP inclinations* were greater during this phase compared to the *Preparation* and 59% of the controls abducted the knee during this phase (*ΔAP inclination 1*,*2 or 3 >* 90°) as compared to 39% of the ACLR group and 65% of the ACLPT group. The *ΔAP inclination* decreased towards the end of the *Action* phase in the ACL groups, resulting in a significantly lower *ΔAP inclination-3 in* ACLPT compared to CTRL (Table 3). A lower Δ*AP inclination-3* also correlated with lower self-reported knee function (rho 0.391, p = 0.002) and higher values of static knee laxity (rho -0.450, p = 0.001).

Side comparisons within the ACL groups showed that for the injured knee, *Duration* was significantly shorter (95% CI injured = 163–189 ms, non-injured = 168–194 ms; 95% CI of mean difference -9 - -0.1 ms; F (1, 49) = 4.2, p = 0.046) and Δ*AP inclination* was significantly smaller towards the end of the phase (95% CI *ΔAP inclination-3*: injured = 73.3–82.9°, non-injured = 81.1–90.7°; 95% CI of mean difference 2–13°; F (1, 39) = 8.3, p = 0.006). The injured side also showed a more anterior Δ*AP position* compared to the non-injured side in the beginning of the *Action* phase (95% CI *ΔAP position -1*: injured = 5.3–22.4 mm, non-injured = -22.4 - -5.3 mm– 90.7°; 95% CI of mean difference 10.2–1.5 mm; F (1, 49) = 7.3, p = 0.009). A significant interaction was found for the first Δ*AP inclination* (F (1, 49) = 6.2, p = 0.02), showing a side difference within the ACLR group (95% CI injured = 70.5–80.0°, non-injured = 75.1–81.6°).

### Principal component analysis of significant kinematic variables

A PCA was performed to further explore the variables that showed significant group differences when comparing non-dominant control knees and injured ACL knees. These variables were *Duration*, *ROM-FE*, *ΔAP inclination -3*, *AP position -1* and *AP position -2*. The PCA resulted in two significant PCs for each phase, where the first PC was mainly explained by *Duration* and *RoM*–*FE* while the second PC was mainly explained by Δ*AP inclination* and Δ*AP position* (Table 4).

**Table 4:** Principal component analyses for significant variables during the *preparation* and *action* phases of the drop jump test.

Selected variable*	*Preparation*^#^		*Action*^#^	
	PC1	PC2	PC1	PC2
*Duration (s)*	0.84	0.20	0.88	0.22
*ROM-FE (°)*	0.82	0.32	0.86	0.24
*ΔAP inc -3 (°)*	-0.78	-0.26	0.12	0.48
*ΔAP pos -1 (mm)*	-0.42	0.74	-0.51	0.66
*ΔAP pos -2 (mm)*	-0.44	0.75	-0.15	0.76
Variance explained (%*)*^$^	47.2	25.8	36.0	26.9

* Variables that showed significant group differences in the mixed model analyses were included in the PCA.

^#^ Principal components (PC’s) with eigenvalue >1 are reported.

^$^ The percentage of variance that each PC explains, expressed in percent of the total covariance matrix

## Discussion

Finite helical axes analysis revealed reduced dynamic knee stability in ACL-injured knees 17–28 years after trauma compared to the contralateral non-injured knees and to persons with asymptomatic knees during *Preparation* and *Action* after a vertical drop jump. Our results further suggest that smaller ranges of knee flexion within shorter landing phases, were used to protect the injured knee during the landing. In addition, despite a two-leg landing, side differences in phase duration (longer *Preparation* and shorter *Action* phases on the injured knee) indicated a leg preference towards the non-injured leg.

### Knee kinematics during preparation

We hypothesised that ACL-injured persons would unload and protect their injured knee during the preparation for the landing after a vertical drop jump, even in the long term after the knee injury. Indeed, during the *Preparation*, persons with a previous unilateral ACL injury had a smaller range of knee flexion in comparisons to asymptomatic controls, and the phase duration was also significantly shorter. An explanation could be that asymptomatic persons are able to perform softer landings with a larger range of knee flexion and still maintain knee translations and rotations within a small, safe range [19]. In contrast, persons with a previous ACL injury may use a stiffening strategy with a smaller range of knee motion to compensate for loss of dynamic stability [20]. This could also explain the significant correlation between a small *RoM-FE* during *Preparation* and low self-rated knee function (Lysholm score).

When analysing the knee FHAs, we found that it had a posterior position during *Action* compared to *Preparation* in all groups. This indicates a posterior translation of the FHA as the knee flexes during the landing. This is in line with a study on sagittal tibial translations during squat exercises, where a posterior translation was found during knee flexion in both healthy and ACL-deficient knees [21]. We found that even though the knee FHA had a posterior position, it was significantly more anterior in ACLPT compared to ACLR;. Greater *ΔAP position* further correlated with a greater static knee laxity according to KT-1000 measurements. This implies that a greater anterior tibial translation occurs in non-reconstructed knees compared to reconstructed knees during drop landings as similarly reported by other researchers [21] who also found a more anterior position of the tibia in ACL-deficient knees during the knee flexion.

### Knee kinematics during action

We expected that excessive abduction-adduction movements and tibial AP translations would occur in the ACL-injured knee during landing after a vertical drop jump. Asymptomatic controls had a longer *Action* phase with a greater knee flexion RoM compared to ACL-injured persons, while an Δ*AP inclination* close to 90° indicated small abduction-adduction movements throughout the phase (86.1–97.3°, Table 3). Conversely, ACL-injured persons had shorter phases with a smaller knee flexion RoM. The Δ*AP inclination* varied more for ACL-injured persons during the drop landing than for controls (Table 3) and became significantly smaller in ACLPT compared to controls towards the end of the *Action* phase. This means that the asymptomatic controls were able to perform a soft landing with larger knee flexion and a stable knee, whereas ACL-injured persons showed smaller flexion angles with a greater adduction-abduction ratio, which confirms our hypothesis.

The ACLPT group also presented a longer *Preparation* and shorter *Action* on the injured knee compared to the non-injured, implying that ACLPT persons weighted more on the non-injured knee during *Action* and instead prolonged the *Preparation* phase on the injured side in order to reduce such weight bearing loading. This agrees with a recent study in which an ACLR group of 17 persons 9 months after reconstruction employed a knee unloading strategy of their involved leg during landing after a drop jump, [11].

### Clinical implications

We found kinematic differences in knee control still 17–28 years after ACL injury. In comparison, a longitudinal study on 14 ACL injured persons showed compensatory changes in the ACL reconstructed knee compared to the non-injured (less flexion, increased external rotation and medial translation of the joint during single-legged hop landing) that decreased and normalised during the first year after ACL reconstruction [22]. Some of the long-term differences in the population groups in our study may have been attributed to differences in sensorimotor function that were present even before the injury occurred, since participants with reduced knee joint motor control may be more susceptible to ACL injuries. Since these tests were performed long time after ACL injury, development of OA was evident in reconstructed knees and even contralateral knees (see [23]), which may have affected the results.

Both ACL groups had different kinematic profiles compared to controls, with smaller *RoM–FE* and *Duration*, probably in order to protect the knee. Based on previous studies on long-term adaptions after ACL injury [3, 4], it was hypothesized that persons treated with reconstruction combined with PT would have higher dynamic knee stability compared to persons treated only with PT. Indeed, ACLPT had a greater Δ*AP position* compared to ACLR and a smaller Δ*AP inclination* compared to controls whereas ACLR did not. One reason could be that ACL-injured legs without reconstruction had larger anterior knee translation and abduction-adduction movements during the drop landing, also implicated by the positive correlation between Δ*AP position* and static knee laxity (KT1000) and the negative correlation between Δ*AP inclination* and KT1000.

A PCA were performed to further explore the kinematic variables that gave significant group differences, in order to analyse which of these variables that influenced the performance the most. This revealed that the FHA variables represent a different aspect of motor control than *RoM-FE* and *Duration*, since two orthogonal PC’s resulted where *RoM-FE* and *Duration* contributed mainly to the first PC, while Δ*AP inclination* and Δ*AP position* mainly contributed to the second PC. *RoM-FE* and *Duration* could be estimated with relatively simple motion capture systems in clinics or by coaches to assess athlete injury risk or when making return to sport decisions. However, in a cross-sectional study on 782 athletes screened during a drop jump test, such discrete kinematic measures could not predict the risk of an ACL injury [10]. A recent review calls for more comprehensive descriptions of movement quality after an ACL injury to facilitate the evaluation of the training and rehabilitation [24]. Detailed kinematic evaluation of landing technique, targeting the knee, offers such an opportunity. The Δ*AP inclination* and Δ*AP position* provide valuable information at a group level about how different tests challenge knee function, even though such measures are less suited to implement clinically. In a wider perspective, research studies that highlight a more efficient treatment strategy could be adopted, while rehabilitation procedures and screening processes in the years following injury could be better tailored to individual needs.

### Methodological strengths and limitations

The kinematic analyses focused on the initial drop landing. While part of the landing process is reflexive in nature, a conscious control and modulation of landing actions builds upon experience [18]. More importantly for the current purpose of this study, in landing-related tasks the ability to dissipate kinetic energy is attributed mainly to knee actions [25]. A strength and main message of our paper is the usefulness of helical axis methods/analysis to determine dynamic knee stability even long after ACL injury. The participants performed only one repetition of the drop jump test which contributed to some data loss and this may have affected the generalizability. The reasons for only one hop were that the drop jump came last in an extensive test battery, and that multiple drop jumps may be overly strenuous particularly in this age group and long after ACL injury when OA is likely to have developed. Indeed, 12 participants performed the jump incorrectly while four chose to refrain from the test. It may be that these four individuals had the lowest knee stability and that the drop jump test is inappropriate to persons with severe knee injuries. Further, females were underrepresented (ACLR 29%, ACLPT 39%, CTRL 40%), which reflects a larger proportion of male individuals in the realm of ACL injuries at the time when the data collection took place.

Finally, it is well-known that soft tissue artefacts influence kinematic calculations, particularly for small joint angular displacements. In order to minimize this influence as much as possible, we used rigid marker clusters and a 6DOF marker model to define the knee motion [26]. We also used a finite HA approach with an interval length of 15° (with a tolerance level of 70%) for the calculations. Calculations of the instant HA was not applicable for the current measurement setup, since HA variables are sensitive to errors when rotation intervals are very small [17]. This finite approach still enabled us to analyse changes in the knee helical axis inclination and position during the preparation and action phases following a vertical drop jump.

## Conclusions

Both ACLR and ACLPT had different kinematic profiles during drop landings compared to persons with asymptomatic knees (smaller *RoM-FE* and *Duration*, greater changes in *ΔAP inclination*), with correlations to worse self-reported knee function (Lysholm score) and greater static laxity (KT-1000). Presumably ACL-injured persons implemented a compensatory strategy to protect the injured knee as evidenced by the observed kinematic side asymmetries (smaller Δ*AP inclination* and a prolonged *Duration*/shortened *Action* phase on the injured compared to the non-injured knee). Briefly, the drop landing is a challenging task for injured knees and may be used to assess dynamic knee control long after an ACL injury.

## Supporting information

S1 File This is the file containing the kinematic variables and scores from questionnaires used for the statistical calculations in this paper. (XLS)
